# Evidence-Based Approaches to Suicide in Older Adults—Collaborative Care

**DOI:** 10.1093/geroni/igaa057.2132

**Published:** 2020-12-16

**Authors:** Michael Schoenbaum

**Affiliations:** National Institute of Mental Health, Bethesda, Maryland, United States

## Abstract

This individual symposium abstract will focus another evidence-based approach to mental health treatment and in older adults, the collaborative care model. Collaborative care is a consultation-based approach in primary care that has been described with multiple clinical trials, with significant benefit for access and treatment. The Prevention of Suicide in Primary Care Elderly: Collaborative Trial (PROSPECT) using the collaborative care model found that those older adults receiving the intervention had a higher utilization of mental health treatment (psychotherapy and/or antidepressant treatment) and had a 2.2 times greater decline in suicidal ideation over 24 months. The authors will describe the utility of using the collaborative care model on the identification of suicidal ideation and subsequent mental health treatment for older adults. The authors will also share about challenges and successes related to collaborative care implementation in healthcare settings for older adults, and relevant policy and financing components for the model.

